# Oxidation of Cefalexin by Permanganate: Reaction Kinetics, Mechanism, and Residual Antibacterial Activity

**DOI:** 10.3390/molecules23082015

**Published:** 2018-08-13

**Authors:** Yajie Qian, Pin Gao, Gang Xue, Zhenhong Liu, Jiabin Chen

**Affiliations:** 1 College of Environmental Science and Engineering, Donghua University, Shanghai 201620, China; yqian@dhu.edu.cn (Y.Q.); pingao@dhu.edu.cn (P.G.); xuegang@dhu.edu.cn (G.X.); zhhl@dhu.edu.cn (Z.L.); 2 School of Environmental Science and Engineering, Suzhou University of Science and Technology, Suzhou 215009, China

**Keywords:** permanganate, water treatment, cefalexin, oxidation, product, antibacterial activity

## Abstract

The oxidation of cefalexin (CFX), a commonly used cephalosporin antibiotic, was investigated by permanganate (PM) in water. Apparent second-order rate constant of the reaction between CFX and PM was determined to be 12.71 ± (1.62) M^−1^·s^−1^ at neutral pH. Lower pH was favorable for the oxidation of CFX by PM. The presence of Cl^−^ and HCO_3_^−^ could enhance PM-induced oxidation of CFX, whereas HA had negligible effect on CFX oxidation by PM. PM-induced oxidation of CFX was also significant in the real wastewater matrix. After addition of bisulfite (BS), PM-induced oxidation was significantly accelerated owing to the generation of Mn(III) reactive species. Product analysis indicated oxidation of CFX to three products, with two stereoisomeric sulfoxide products and one di-ketone product. The thioether sulfur and double bond on the six-membered ring were the reactive sites towards PM oxidation. Antibacterial activity assessment indicated that the activity of CFX solution was significantly reduced after PM oxidation.

## 1. Introduction

The β-lactam antibiotics, including penicillins and cephalosporins, are among the most extensively used antibiotics in human and veterinary medicine [1]. After consumption, a large portion of the administered dosages are excreted unchanged, thus a substantial amount of β-lactam antibiotics is expected to be released into the environment. Because β-lactam antibiotics developed at early stage, e.g., penicillin G, are susceptible to acid/β-lactamase catalyzed hydrolysis, their occurrence in the environment is scarcely reported. However, with the development of pharmacology, some β-lactam antibiotics are designed to be acid-stable and β-lactamase resistant, thus they are frequently detected in the environment [2]. For example, cefalexin (CFX), one of the most frequently used first generation cephalosporins, is relatively stable in aquatic environments. It has been widely detected in different water matrices, such as wastewater, surface water and even coastal water with the concentration up to μg·L^−1^ [3,4,5]. The residual antibiotics in the environment could lead to adverse effects on non-target organisms, pose risk to drinking water supplies and increase the bacterial resistance [6]. Cephalosporins have the most prevalence of antibacterial resistance in pathogens according to the WHO 2014 report on antimicrobial resistance surveillance [7]. Therefore, it is imperative to develop effective strategies to eliminate CFX in the water environment. 

Chemical oxidation processes involving permanganate (PM, Mn(VII)) are promising technologies for eliminating contaminants in the water environment. Compared with other conventional oxidation processes, PM oxidation process is relatively cost-effective and less likely to generate disinfection byproducts [8]. As an oxidant, PM exhibits a relatively high redox potential (i.e., E0 = 0.59–1.68 V) and is effective for oxidizing a variety of inorganic and organic compounds, such as dissolved iron and manganese, odors and pharmaceuticals [9,10]. PM is always regarded as a selective oxidizing agent which exhibits highly variable rates to various contaminants. Generally, the second-order reaction rate constants between PM and organic contaminants are the range of 10^−5^–10^3^ M^−1^·s^−1^ [11,12], which are relatively lower than those for the hydroxyl or sulfate radicals based oxidation (*k*″, 10^9^–10^10^ M^−1^·s^−1^). Recently, Sun et al. reported that the activation of PM by bisulfite (BS) can significantly enhance the oxidation of organic contaminants [13] and Mn(III) is deemed as the major reactive species involved in the oxidation process. However, it is still unclear whether PM exhibits reactivity towards CFX. As discussed below, PM was found to show considerable reactivity towards CFX, and the presence of BS could significantly enhance PM-induced oxidation of CFX.

As an oxidant, PM is expected to react with the moieties with available electrons during the oxidation of organic contaminants. In the molecular structure of CFX, the primary amine, and the thioether sulfur and double bond on the six-membered ring are electron-rich moieties, and thus they are potentially susceptible to electrophilic attack by the oxidants and lose electrons in the reaction. For example, the phenylglycine primary amine are the potentially reactive sites in the oxidation of CFX by Cu(II) [1] and ferrate(VI) [14]. The thioether sulfur on the six-membered ring was reported to be reactive to peroxymonosulfate [15] and sulfate radicals [16], while the double bond on the ring is susceptible to ozone oxidation [2]. The reactivity of PM with the above electron-rich moieties are still unknown, thus need to be further evaluated. Although CFX is susceptible to oxidative degradation by different oxidants, the products might still exhibit antibacterial activity, and thus pose potential risk to aquatic environment [17]. For example, biologically active products were found to be generated in the reactions of PG and CFX with ozone [2]. Ferrate(VI) oxidation is effective to lower the antibacterial activities of β-lactams, but ~24% residual activity is still retained for the treated CFX [14]. Advanced oxidation processes (AOPs) are among the most effective processes for destroying micro-pollutants. The increase of antibacterial activities is reported in the hydroxyl radical-based AOPs treated water samples, such as UV/H_2_O_2_ and photo-Fenton processes [18,19]. Therefore, it is essential to evaluate the antibacterial activity of the transformation products of CFX by PM oxidation.

In this work, we investigated the oxidation of CFX by PM. The objectives were: (1) to investigate the reactivity of PM towards CFX, and determine their reaction kinetics; (2) to evaluate the impact of reaction parameters and water matrices on PM-induced oxidation of CFX, and then assess the promoting effect of BS on CFX oxidation by PM; and (3) to identify the reactive sites on CFX towards PM oxidation, and then evaluate the antibacterial activity of the transformation products. As CFX shares the basic structure with other cephalosporin antibiotics, the findings of this work can provide important references for other cephalosporin antibiotics oxidation by PM.

## 2. Results and Discussion

### 2.1. Oxidation of CFX by PM

**Figure 1:**
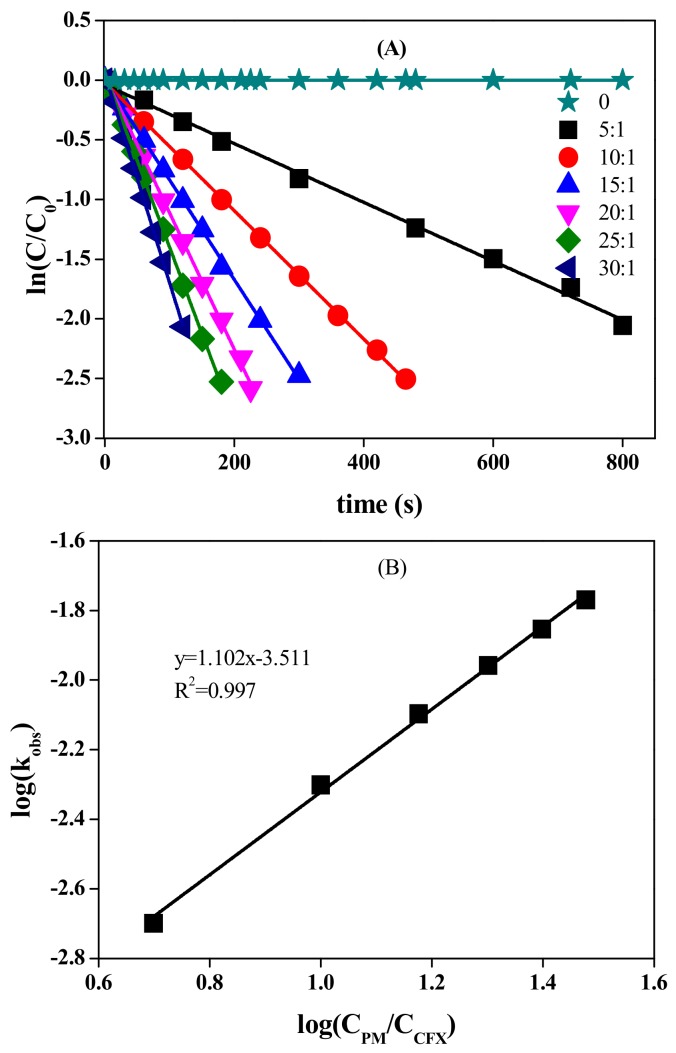
Effect of PM concentration on the oxidation of CFX (**A**), and log (*k_o__bs_*) vs. log (*C_PM/CFX_*) (**B**). [CFX] = 40 μM, pH 7.0. Note: the ratio is PM/CFX.

CFX possesses several electron-rich moieties, e.g., double bond and thioether sulfur on the six-membered ring, thus it is potentially reactive towards oxidants, such as PM. Indeed, rapid oxidation of CFX was observed when PM was present in the solution. For example, CFX was stable at neutral pH condition, but was almost completely degraded within 480 s after addition of 400 μM PM (Figure 1). The oxidation of CFX followed the pseudo first-order kinetics, with the linear relationship (R^2^) higher than 0.995. The observed first-order rate constant (*k_obs_* in Equation (1)) was calculated to be 0.005 s^−1^. The effect of PM concentrations on CFX oxidation was further evaluated at pH 7.0. The results showed that CFX oxidation increased when PM/CFX ratio increased from 5 to 30 (Figure 1A). The degradation of CFX followed first-order kinetics at different concentrations of PM, and the *k_obs_* values increased from 0.002 to 0.014 s^−1^ as the ratio increased from 5 to 30. The log (*k_obs_*) was linearly increased with log (*C_PM_*/*C_CFX_*), with the slope close to 1. Hence, *k_obs_* was first-order with respect to PM concentration. Apparent second-order rate constant (*k*_2,*app*_ in Equation (2)) was subsequently calculated to be 12.71 ± (1.62) M^−1^·s^−1^ by dividing *k_obs_* by the concentration of PM. The obtained *k*_2,*app*_ value of CFX with PM was comparable to those with peroxymonosulfate [15], but was smaller than those with ozone [2], ferrate(VI) [14] and radical species, e.g., sulfate radicals [20].

(1) $$-\frac{d[\mathrm{CFX}]}{\mathrm{dt}}=k_{obs}\times [\mathrm{CFX}]$$

(2) $$-\frac{d[\mathrm{CFX}]}{\mathrm{dt}}=k_{2,app}\times [\mathrm{CFX}]\times [\mathrm{PM}]$$

### 2.2. Effects of pH

**Figure 2:**
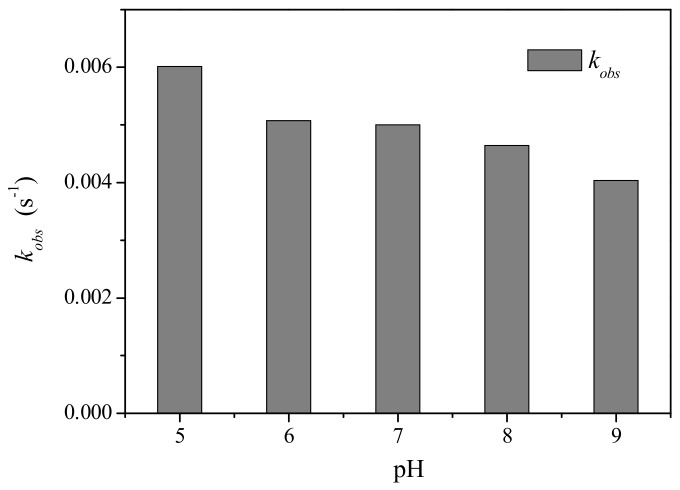
Effect of pH on the *k_obs_* of CFX by PM oxidation. [CFX]_0_ = 40 μM, [PM]_0_ = 400 μM.

The solution pH has been regarded as an important factor influencing oxidation of various organic contaminants by PM. Hence, the impact of pH was examined for PM-induced oxidation of CFX at pH 5.0–9.0, with the result shown in Figure 2. The oxidation of CFX by PM was pH dependent, and lower pH was favorable for PM-induced oxidation of CFX. Specifically, the *k_obs_* of CFX gradually decreased from 0.006 to 0.0041 s^−1^ when pH increased from 5 to 9. Generally, the redox potential of PM decreased with increasing pH, i.e., acidic pH was favorable for enhancing the oxidation capacity of PM [21]. Hence, the faster oxidation of CFX by PM at lower pH might be attributed to the higher oxidation capacity of PM. This result was consistent with the previous reports that PM-induced oxidation of diclofenac decreased with the elevated pH [22]. However, the oxidative reactivity of PM with some contaminants, e.g., lincomycin and sulfamethoxazole, increased at elevated pH [23,24], whereas the oxidation of dichlorvos by PM was found to be unaffected by the solution pH [25]. Hence, pH impact on PM-induced oxidation of organic contaminants was complicated, which was not only dependent on the oxidation capacity of PM, but also on the structure of target contaminants. 

The pH-dependent reactivity of PM towards contaminants was always related to the p*K_a_* value of contaminants. For example, the reactivity of PM towards bisphenol A (BPA) was significantly dependent on pH when the solution pH was close to the p*K_a_* value of BPA [26]. When the solution pH was far away from the p*K_a_* value of carbamazepine (CBZ), the reactivity of PM towards CBZ was almost unaffected by the pH variation [26]. pH could affect the protonation/deprotonation of some functional group, e.g., primary amine, and thus influenced its electron density and susceptibility to PM oxidation. The p*K_a_*_2_ of CFX is 6.86, thus the primary amine on the side chain of CFX was deprotonated at alkaline pHs and possesses higher electron density than the protonated ones at pH 5. If primary amine was the reactive site on CFX, faster degradation of CFX by PM could be expected at alkaline pHs, which was opposite to the results in this work. Therefore, the primary amine on the side chain might be not the reactive site for PM oxidation, which is further elucidated in the following sections.

### 2.3. Effects of Water Matrices

**Figure 3:**
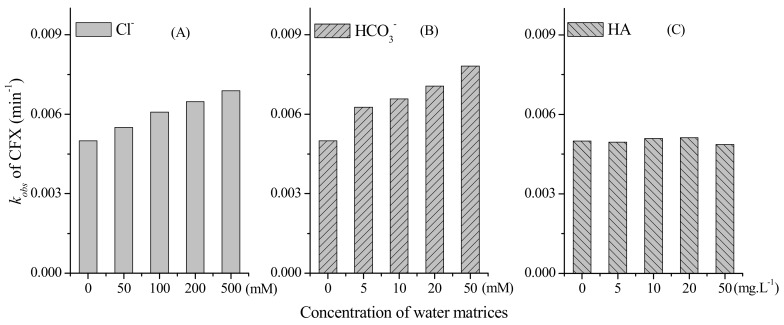
Degradation rate constants (*k_obs_*) of CFX under different concentration of: (**A**) Cl^−^; (**B**) HCO_3_^−^; and (**C**) NOM. [CFX]_0_ = 40 μM, [PM]_0_ = 400 μM, pH 7.

The water matrices, e.g., the inorganic and organic solute, might affect the oxidation of target organic contaminants during PM oxidation. Hence, we further evaluated the effect of common ions, e.g., Cl^−^ and HCO_3_^−^, on PM-induced CFX oxidation. As shown in Figure 3A, the calculated *k_obs_* of CFX oxidation increased with the increasing concentration of Cl^−^ from 0 to 500 mM. Similarly, the presence of HCO_3_^−^ promoted the oxidation of CFX by PM, and the rate constants also increased when HCO_3_^−^ concentrations increased from 0 to 50 mM (Figure 3B). This result was consistent with a previous report that the common ions, e.g., HCO_3_^−^, exhibited strong accelerating effect on BPA oxidation by PM, but the mechanism was still ambiguous and need further investigation [27].

We further investigated the impact of HA on the oxidation of CFX by PM, and the result showed that PM-induced oxidation of CFX was not affected by various concentrations of HA (Figure 3C). As a common matrix in the real waters, the effect of HA on PM-induced oxidation of organic contaminants have been extensively evaluated, and its impact was complicated. HA was regarded as a reductant to inhibit the oxidation of SMX by PM [23], whereas it was also reported to promote the oxidation of some organic contaminants when the concentration of HA was lower than PM [28]. In some previous reports, the impact of HA on the oxidation of contaminants were dependent on HA concentration and source. For example, HA could accelerate the oxidation of phenol by PM when low concentration of HA was present in the solution, but gradually inhibited phenol oxidation as HA concentration increased [29]. At the constant concentration of HA, commercial HA and HA extracted from soils exhibited different impact on the contaminant degradation, which could be attributed to their different functional groups [30]. In another study, HA also exhibited negligible effect on the PM-induced oxidation of contaminants [9], which was consistent with the result in this work. Although the examined HA exhibited negligible effect, a wider diversity of HA sources need to be evaluated on whether HA influences reaction between CFX with PM [27].

PM-induced oxidation of CFX was also evaluated in the real water sample. Four hundred micromolars of PM and 0.2 μM of CFX were spiked into wastewater (WW) sample (water quality is shown in Table S1), which was maintained at pH 7 with PB (10 mM). Before spiking, CFX was confirmed to be negligible in the WW sample. Results showed that significant degradation of CFX was observed in WW without addition of PM (Figure S1). The β-lactam ring in CFX was susceptible to breakage catalyzed by metal ions, alkali and other water matrices [0], hence CFX degradation in WW was significant even without spiking PM. After addition of PM, the degradation of CFX was significantly enhanced, and PM-induced degradation of CFX in WW was slightly slower than that in the DI water (Figure S1). In the real WW sample, although the anions, e.g., HCO_3_^−^ and Cl^−^, could promote PM-induced oxidation of CFX, the water matrix, e.g., reduced substances, might also consume PM, thus lowering the degradation of CFX by PM. Overall, PM-induced oxidation of CFX was significant in the real wastewater matrix, and thus PM was an efficient oxidant to eliminate CFX in the real WW samples.

### 2.4. Impact of BS on PM-Induced Oxidation of CFX

BS has been reported to significantly accelerate the oxidation of organic contaminants, e.g., phenol, by PM. Hence, we further evaluated the oxidation of CFX in the PM/BS system. As shown in Figure S2, degradation of CFX was negligible in the presence of 600 μM BS alone, while about 30% of CFX was degraded within 15 min by 80 μM PM. However, complete degradation of CFX was achieved within 10 min in the combination of PM and BS. The results indicated that PM and BS had synergistic effect on the oxidation of CFX. Indeed, BS was regarded as an efficient activator for PM to generate a reactive intermediate, i.e., Mn(III), which could oxidize organic contaminants at an extremely rapid rate. In PM/BS system, the rapid oxidation of CFX was also likely induced by the reactive Mn(III) generated from the reduction of PM by BS.

**Figure 4:**
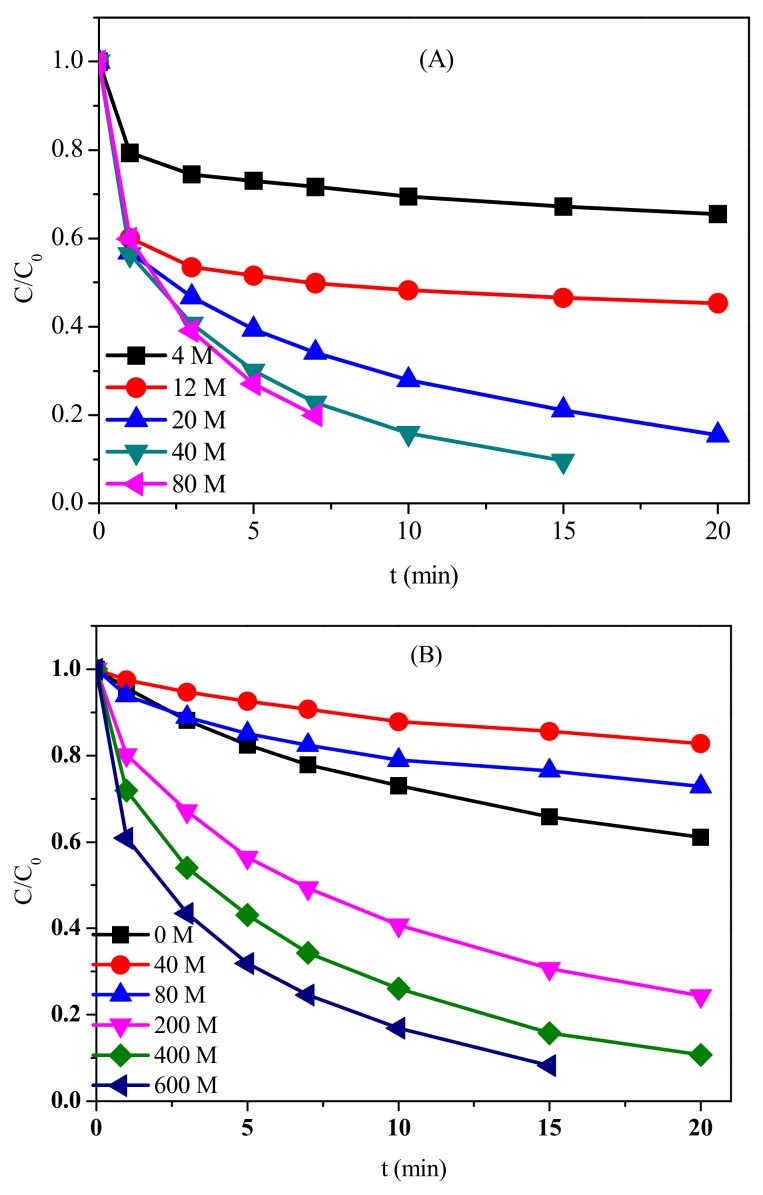

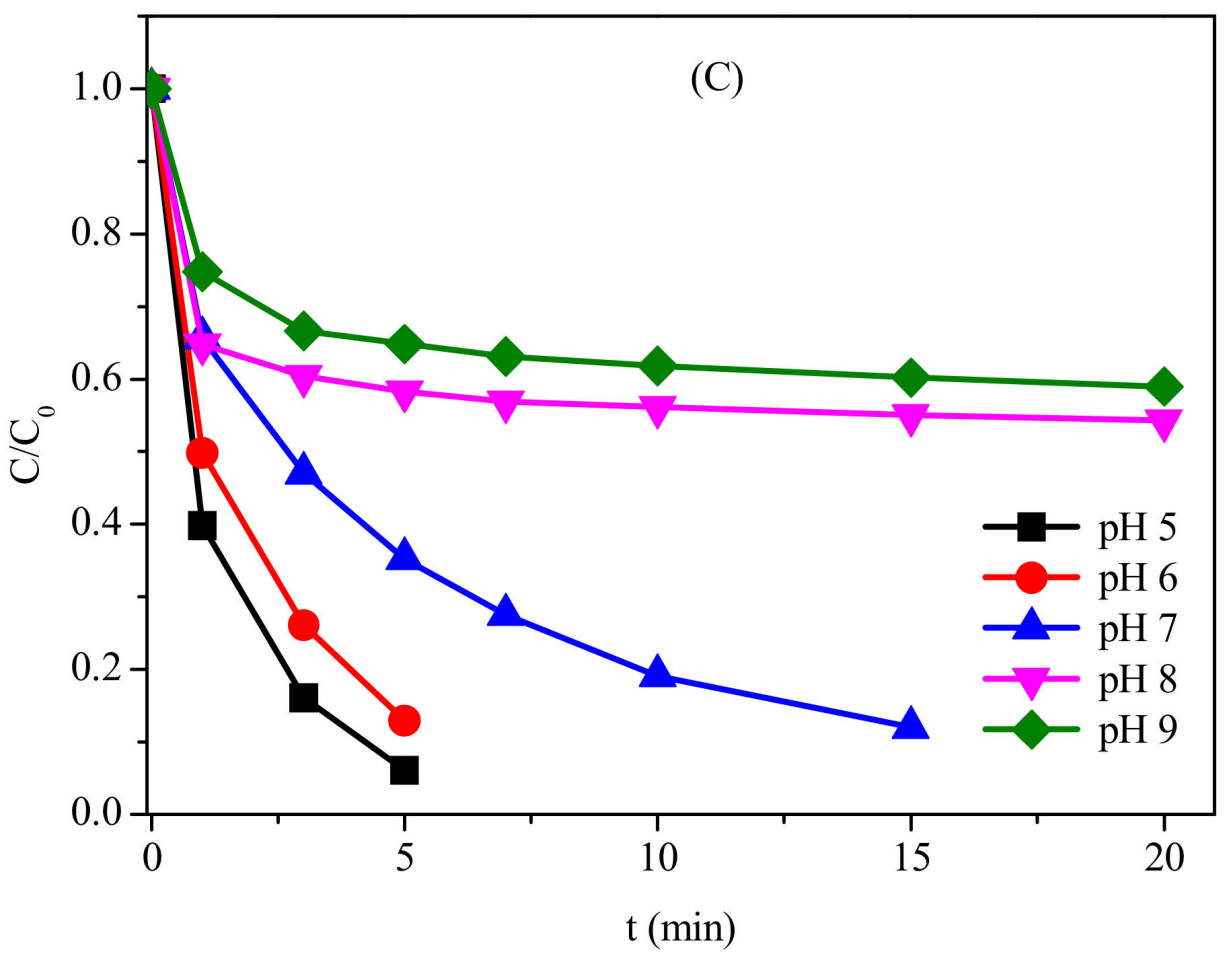
Effect of PM concentrations (**A**); BS dosages (**B**); and pH (**C**) on the oxidation of CFX in PM/BS system. [CFX]_0_ = 40 μM; (**A**) [BS]_0_ = 600 μM, pH = 7; (**B**) [PM]_0_ = 40 μM, pH = 7; and (**C**) [PM]_0_ = 40 μM, [BS]_0_ = 600 μM.

The impact of different dosages of PM and BS on the oxidation of CFX was further investigated (Figure 4A). In the absence of BS, the oxidation of CFX increased from 4% to 65% after 20 min when PM dosage increased from 2 μM to 80 μM (Figure S3). In the presence of 600 μM BS, CFX oxidation increased from 35% to 75% after 20 min with the increasing dosage of PM from 2 μM to 20 μM. Complete degradation of CFX could be achieved when PM further increased to 40 μM. However, the degradation of CFX could not be further enhanced when PM increased to 80 μM. With the increasing dosage of PM, more Mn(III) reactive species could be generated to oxidize CFX. When high dosage of PM was present, PM could consume the Mn(III), thus the degradation of contaminant could not be further increased.

Compared to the impact of PM concentration, effect of BS concentration on the oxidation of CFX was more complicated. In the presence of 40 μM PM, the oxidation of CFX was slightly inhibited in the presence of low BS dosage, but was significantly promoted when high concentration of BS was present (Figure 4B). For example, the degradation efficiency of CFX within 20 min decreased from 39% (PM alone) to 17% and 25% when 40 μM and 80 μM BS were present, respectively. However, almost complete degradation of CFX was achieved within 20 min and 15 min in the presence of 400 μM and 600 μM BS, respectively. When low concentration of BS was present in PM/BS system, the generated Mn(III) could be consumed by PM, thus reducing the effective oxidants (i.e., PM and Mn(III)) responsible for CFX degradation. When the concentration of BS increased to 200 μM or even higher, almost all PM was transformed to Mn(III), thus the reactive Mn(III) was the dominant oxidant in the reaction system. 

pH also had a significant impact on CFX oxidation in PM/BS system. As shown in Figure 4C, CFX degradation was rapid at pH 5.0 and decreased as pH increased from 5.0 to 9.0. As the generated Mn(III) is unstable and prone to disproportionate under alkaline conditions [13], higher pH was unfavorable for the oxidation of CFX in PM/BS system. Furthermore, the speciation of BS is strongly influenced by solution pH (HSO_3_^−^ ⇌ SO_3_^2^^−^ + H^+^, p*K_a_* = 7.2), which will convert to less reactive sulfite under alkaline conditions [31]. Hence, acidic condition was favorable for CFX oxidation.

### 2.5. Product Analysis

To identify the potential reaction site of CFX towards PM oxidation, we subsequently analyzed the oxidation products of CFX by PM. Transformation products of CFX by PM were analyzed by LC/MS/MS. Two products with molecular weight (MW) of 363, and one product with MW of 379 were generated after oxidation of CFX (MW: 347) by PM (Figure S4–S6). The MW 363 (M + 16) and 379 products (M + 32) were likely generated via the addition of one or two oxygen atoms on the molecule of CFX, respectively. The two MW 363 products (363a and 363b) might be isomers since they have different LC retention time but similar MW and MS fragment patterns. The M + 16 products were also previously reported as the main oxidation products of CFX by peroxymonosulfate [15], ozone [2], ferrate (VI) [14], and sulfate radical [32]. They were identified as stereoisomeric CFX-(R)-sulfoxide and CFX-(S)-sulfoxide. We subsequently analyzed the oxidation products of CFX by peroxymonosulfate with the analytical methods in this work. The result indicated that CFX-(R)-sulfoxide and CFX-(S)-sulfoxide generated by peroxymonosulfate matched the 363a and 363b products in LC retention time and MS fragment patterns. Hence, the 363a and 363b products were verified as CFX-(R)-sulfoxide and CFX-(S)-sulfoxide, respectively. Based on the MS spectrum, 379 product contains the fragment of *m*/*z* 106, which is characterized by the intact primary amine moiety on the side chain. Hence, the two oxygen atoms were most likely added on the double bond on six membered ring, which was also proposed in the oxidation of CFX by ozone [2]. 

**Figure 5:**
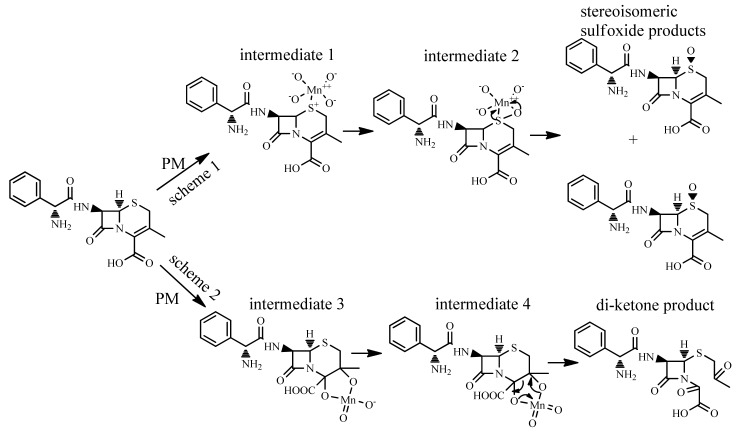
Transformation products of CFX by PM.

For the sulfoxide products, the thioether sulfur on the six-membered ring of CFX was the reaction site for PM oxidation. Indeed, the thioether sulfur was susceptible to oxidative transformation by various oxidants, e.g., peroxymonosulfate [15], ferrate(VI) [14] and sulfate radicals [32]. The CFX-sulfoxide products were generated by the electrophilic attack of the positively charged Mn atom in PM on the thioether sulfur to form Mn-sulfur intermediate 1. Subsequently, a pair of electrons of sulfur were donated to a d-orbital of Mn, inducing the formation of a coordinate covalent intermediate 2, which were further decomposed to sulfoxide products by rearrangement [33] (Figure 5, scheme 1). For the 379 product, the double bond on the six-membered ring was the reaction site for PM oxidation. The positively charged Mn electrophile attacked the unsaturated C=C bond on the ring, and then forming cyclic hypomanganate(V) ester (intermediate 3) via an activated organometallic complex. Afterwards, the cyclic manganite(VI) ester (intermediate 4) was generated from the cyclic hypomanganate(V) ester by the oxidation of PM, and subsequently hydrolyzed to generate di-ketone-containing product [33] (Figure 5, scheme 2).

### 2.6. Antibacterial Activity Assessment

**Figure 6:**
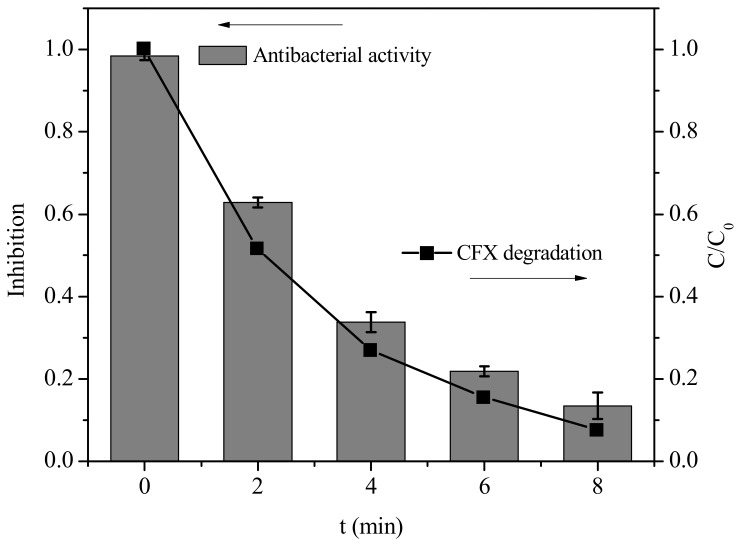
Growth inhibition of *Escherichia coli*. [CFX] = 40 μM, [PM] = 400 μM, and pH = 7 (10 mM PB). Error bars represent standard deviations of data from triplicate samples.

Although PM was effective to oxidize CFX, the β-lactam ring still existed in the transformation products. To investigate whether the products possess biological activity, we further evaluated the antibacterial activity of the transformation products of CFX by PM with *E. coli* as the tested bacteria. As shown in Figure 6, the growth of *E. coli* could be completely inhibited in the presence of 40 μM CFX. After addition of PM, the antibacterial activity of CFX solution gradually decreased along with the gradual oxidation of CFX. Specifically, the growth inhibition was reduced to 13% after 8 min when about 92% of initial CFX was oxidatively degraded in the presence of PM. This result indicated that the biological activity of CFX could be significantly reduced after treatment of PM. Various oxidants have been investigated to remove the antibacterial activity of CFX. For example, peroxymonosulfate [15], ozone [34] and ferrate(VI) [14] were found to significantly eliminate the biological activity of CFX. However, the products of β-lactam antibiotics (e.g., AMX) with UV/TiO_2_ still exhibited significant activity to *Enterococcus faecalis* (ATCC 14506). The results in this work indicated that PM was effective to eliminate the biological activity of CFX.

## 3. Materials and Methods

### 3.1. Chemicals

Cefalexin (CFX, ≥99%) was purchased from Sigma-Aldrich (St. Louis, MO, USA). Potassium permanganate (PM, ≥99.5%) and sodium bisulfite (BS, ≥99.9%) were obtained from Aladdin (Shanghai, China). The HPLC grade of phosphoric acid and methanol were obtained from Sigma-Aldrich. Humic acid (HA) was purchased from ANPEL Laboratory Technologies Inc. (Shanghai, China). Other chemicals and reagents used were in analytical grade or higher, and obtained from Sigma-Aldrich (St. Louis, MO, USA) or Aladdin (Shanghai, China). All chemicals were used as received without any further purification. Reagent grade deionized (DI) water was produced by a Milli-Q Ultrapure Gradient A10 purification system (Millipore, Burlington, MA, USA).

### 3.2. Real Water Samples

Wastewater (WW) after biological treatment was sampled from a municipal wastewater treatment plant located in the south region of China. Samples were filtered through 0.45 μm glass fiber filters and then stored under 4 °C before use. The water parameters of WW are present in Table S1.

### 3.3. Experimental Setup

Batch experiments were conducted in 100 mL amber serum bottles open to the air. The reaction solution in the bottles were constantly mixed by a magnetic stirring. Except where mentioned, phosphate buffer (PB, 10 mM) was used to control the solution pH at 7. PM (400 μM) were added into the solution containing CFX (40 μM) and PB to initiate the reaction. The pre-determined concentration of Cl^−^, HCO_3_^−^ or HA was added into the above reaction system to evaluate their impact on CFX degradation. BS (600 μM) was added in the solution containing PM (40 μM), CFX (40 μM), and PB to evaluate the effect of BS. To investigate the pH impact, the solution was controlled at pH 5–9 with 10 mM PB. Samples were periodically withdrawn and supplemented with excess sodium thiosulfate to quench reactive species. The quenched samples were stored at 2 mL amber vial at 4 °C and analyzed within 24 h. All experiments were conducted in duplicates or more.

### 3.4. Analytical Methods

CFX was analyzed by an Agilent 1200 series HPLC system equipped with a UV diode-array detector (DAD). Samples were separated using a Zorbax Extend-C18 column (4.6 × 250 mm, 5 μm) and detected at 262 nm. Isocratic elution was employed using 10 mM H_3_PO_4_ solution and methanol (70:30, *v*/*v*) with a flow rate of 1 mL/min. 

The transformation products were separated on HPLC system (Utimate 3000, Dionex, Sunnyvale, CA, USA) with a C18 column (2.1 × 150 mm, 5 μm), and analyzed with a triple quadrupole mass spectrometry (TSQ Quantum Ultra EMR, Thermo Fisher Scientific, Waltham, MA, USA). The mobile phase consisted of (A) 0.1% formic acid in water and (B) acetonitrile at a flow rate of 0.2 mL/min: 5% B was kept for 2 min, then ramped to 30% B over 10 min, kept for 10 min. The mass spectrometer was operated at positive electrospray ionization (ESI+). Then, product-full-scan mode was used to obtain sufficient fragment information for structure elucidation.

### 3.5. Bacterial Growth Inhibition Bioassays 

The antibacterial activity of CFX before and after treatment by PM was determined with *E. coli* (ATCC 25,922) as reference strains. Briefly, 4 mL aliquots were taken at the predetermined time (i.e., 0, 2, 4, 6, and 8 min), and immediately quenched by Na_2_S_2_O_3_. Then, the sample was subsequently inoculated with 1 mL of a broth *E. coli* culture (1 × 10^6^ CFU mL^−1^), which was incubated overnight in sterile Mueller–Hinton broth at 37 °C. The samples were further incubated for 8 h at 37 °C on a shaking incubator rotating at 170 rpm. Control experiments with Na_2_S_2_O_3_ or DI water dosing 1 mL of *E. coli* broth culture were also conducted. The growth of *E. coli* was found to be unaffected by Na_2_S_2_O_3_. After the incubation, the bacterial growth was evaluated by determining the optical density (OD) at 625 nm on a UV-vis spectrophotometer (Mapada UV-1600 PC), and then comparing this value to the initial OD value of each sample. The OD value was subsequently converted to growth inhibition, I, according to

I = (A_0_ − A)/A_0_

where A_0_ represents the maximum absorbance for control, which corresponds to uninhibited culture growth. The values of I vary from 0 (null inhibition) to 1 (complete inhibition). All experiments were performed in triplicates, and the results were reported as the mean with standard deviations.

## 4. Conclusions

PM exhibits high performance in the oxidative degradation of CFX. Apparent second-order rate constant of the reaction between CFX and PM was calculated to be 12.71 ± 1.62 M^−1^·s^−1^. Lower pH was favorable for PM-induced oxidation of CFX. PM-induced CFX oxidation could be promoted in the presence of Cl^−^ and HCO_3_^−^, and the promoting effect increased with the increasing concentration of coexisted ions, whereas HA had no effect on the oxidation of CFX by PM. Oxidation of CFX by PM was also significant in the wastewater matrix. The presence of BS could significantly accelerate the oxidation of CFX by PM. Product analysis indicated that two products with MW of 363 and one product with MW of 379 were the primary products of CFX by PM oxidation. The thioether sulfur and double bond on the six-membered ring were proposed as the main reaction sites in CFX towards PM oxidation. The antibacterial activity of the transformation products was significantly reduced after treatment of CFX by PM. Therefore, PM oxidation was an effective approach to significantly eliminate CFX and its biological activity.

## Supplementary Materials

The supplementary materials are available online.
