# Supplementary material for: Oxidation of Cefalexin by Permanganate: Reaction Kinetics, Mechanism, and Residual Antibacterial Activity
Source: Molecules. 2018 Aug 13;23(8):2015. doi: 10.3390/molecules23082015 (PMC6222860; doi:10.3390/molecules23082015)
Supplement: Supplementary file 1 [file molecules-23-02015-s001.pdf]

Supporting Information

Oxidation of cefalexin by permanganate: Reaction kinetics, mechanism, and residual antibacterial activity

Yajie Qian [1], Pin Gao [1], Gang Xue [1], Zhenhong Liu [1], Jiabin Chen [2,*]

[1] College of Environmental Science and Engineering, Donghua University, Shanghai, 201620, P. R. China

[2] School of Environmental Science and Engineering, Suzhou University of Science and Technology, Suzhou, 215009, P. R. China

* Corresponding Author. Phone: +86 0512 68096895; Fax: +86 0512 68096895.

Email: chenjiabincn@163.com (Jiabin Chen).

Journal: Molecules

Date Prepared: July 20, 2018

Figures: S1-S6

Tables: S1

Table S1. Characteristics of the wastewater samples

pH	DOC	Cl^-	PO_4^{3-}	Alkalinity
7.31	21.2	145	0.73	42.1

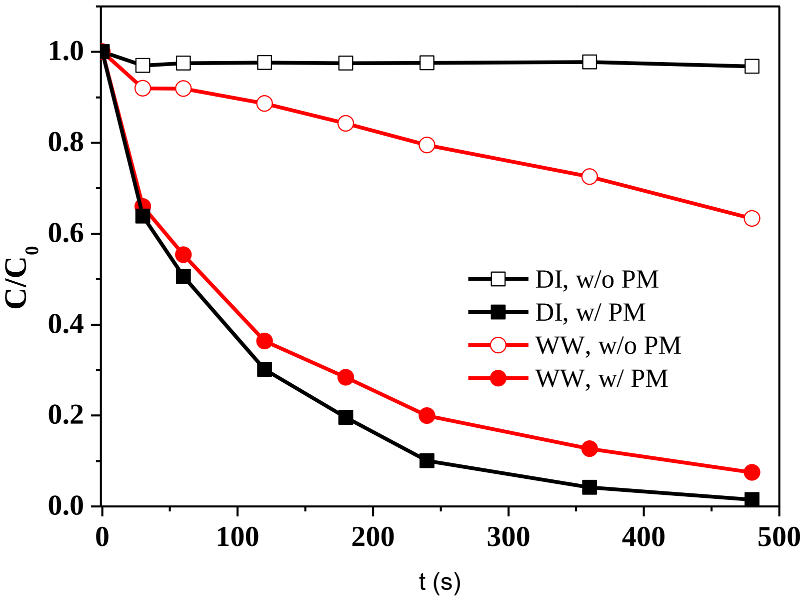

Figure S1. PM-induced degradation of CFX in the real water matrix. Conditions: [CFX] = 0.2 μM; [PM] = 400 μM; and pH = 7 (10 mM PB). Note: DI: deionized water, WW: wastewater, w/: with, w/o: without.

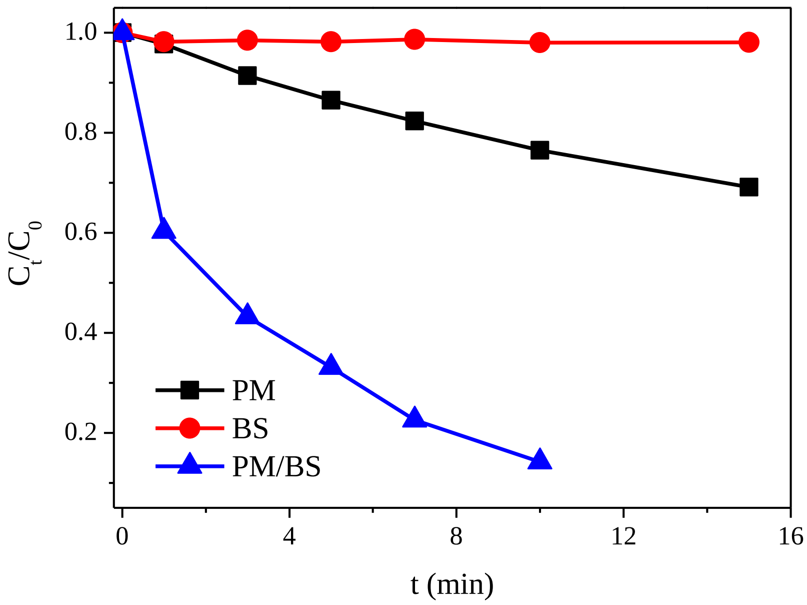

Figure S2. The degradation efficiency of CFX by PM and PM/BS. [PM] = 40 μM, [BS] = 600 μM, pH = 7.

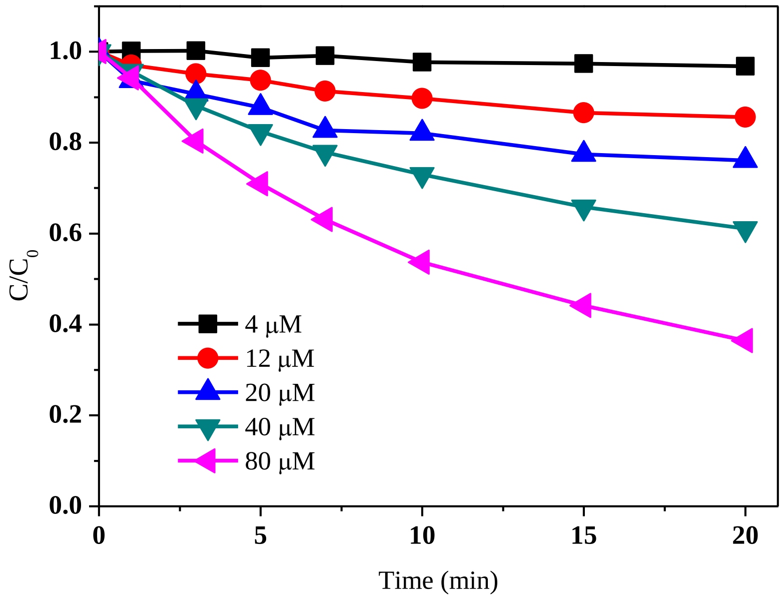

Figure S3. Effect of PM concentration on CFX oxidation by PM. [CFX] = 40μM, pH = 7.

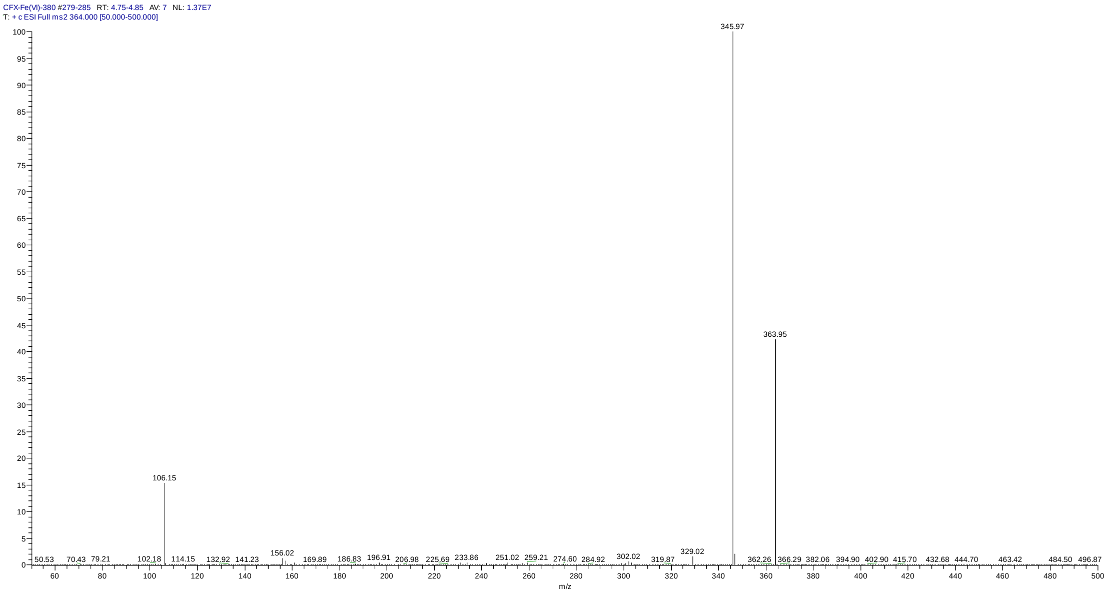

Figure S4. ESI MS^2 spectrum acquired for 363a.

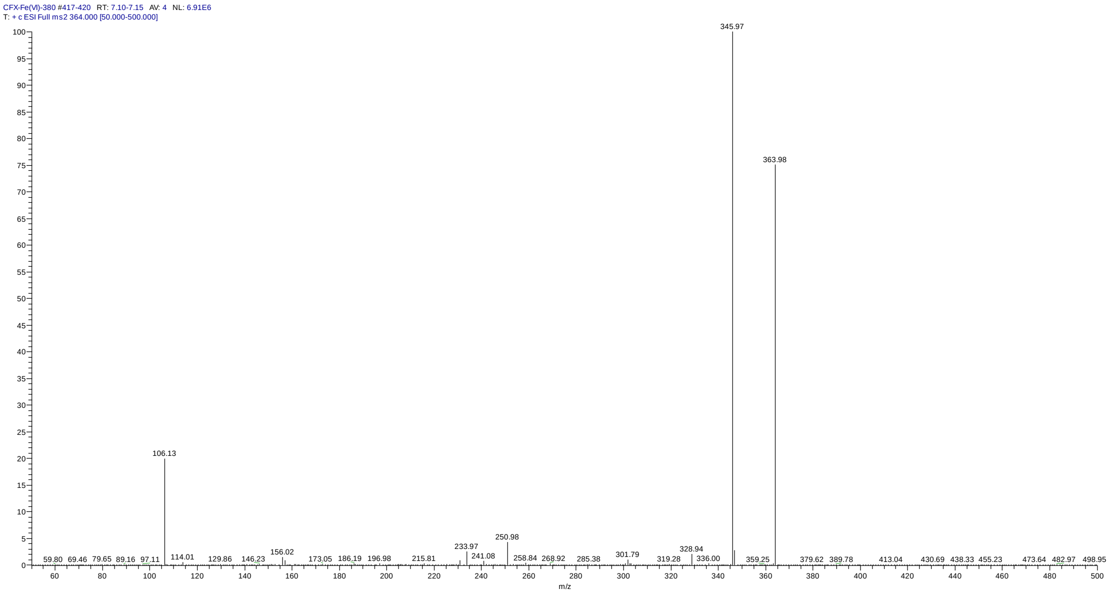

Figure S5. ESI MS^2 spectrum acquired for 363b.

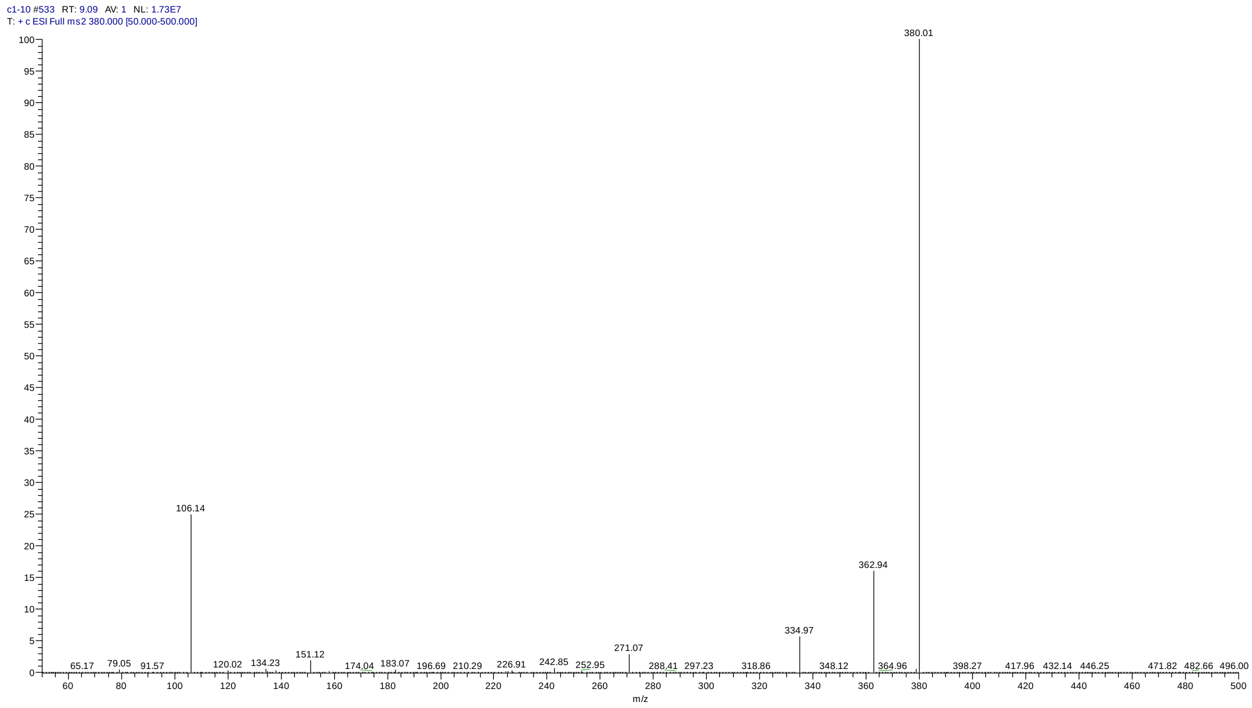

Figure S6. ESI MS^2 spectrum acquired for 379.
